# Recent Advances in Nanostructured Inorganic Hole-Transporting Materials for Perovskite Solar Cells

**DOI:** 10.3390/nano12152592

**Published:** 2022-07-28

**Authors:** Dingyan Huang, Huimin Xiang, Ran Ran, Wei Wang, Wei Zhou, Zongping Shao

**Affiliations:** 1 State Key Laboratory of Materials-Oriented Chemical Engineering, College of Chemical Engineering, Nanjing Tech University, Nanjing 210009, China; 202161204273@njtech.edu.cn (D.H.); 202062104009@njtech.edu.cn (H.X.); ranr@njtech.edu.cn (R.R.); zhouwei1982@njtech.edu.cn (W.Z.); 2 WA School of Mines: Minerals, Energy and Chemical Engineering, Curtin University, Perth, WA 6845, Australia

**Keywords:** perovskite solar cells, nanostructure, inorganic hole-transporting materials, stability, power conversion efficiency

## Abstract

Organic-inorganic halide perovskite solar cells (PSCs) have received particular attention in the last decade because of the high-power conversion efficiencies (PCEs), facile fabrication route and low cost. However, one of the most crucial obstacles to hindering the commercialization of PSCs is the instability issue, which is mainly caused by the inferior quality of the perovskite films and the poor tolerance of organic hole-transporting layer (HTL) against heat and moisture. Inorganic HTL materials are regarded as promising alternatives to replace organic counterparts for stable PSCs due to the high chemical stability, wide band gap, high light transmittance and low cost. In particular, nanostructure construction is reported to be an effective strategy to boost the hole transfer capability of inorganic HTLs and then enhance the PCEs of PSCs. Herein, the recent advances in the design and fabrication of nanostructured inorganic materials as HTLs for PSCs are reviewed by highlighting the superiority of nanostructured inorganic HTLs over organic counterparts in terms of moisture and heat tolerance, hole transfer capability and light transmittance. Furthermore, several strategies to boost the performance of inorganic HTLs are proposed, including fabrication route design, functional/selectively doping, morphology control, nanocomposite construction, etc. Finally, the challenges and future research directions about nanostructured inorganic HTL-based PSCs are provided and discussed. This review presents helpful guidelines for the design and fabrication of high-efficiency and durable inorganic HTL-based PSCs.

## 1. Introduction

Nowadays, the energy supply of our society is mainly reliant on the combustion of fossil fuels, which is limited by the serious CO_2_ emissions, inferior energy conversion efficiency and non-renewable nature of fossil fuels [1,2]. Thus, the exploration and utilization of renewable and clean energy resources have received rapidly increasing interest to meet the ever-grown energy demand and resolve environmental issues [3,4,5]. Among various renewable energies, solar energy has attracted extensive attention because of its abundance and clean nature. At present, the utilization of solar energy can be mainly categorized into photovoltaic (solar cells), photocatalysis and photothermal technologies [6,7,8,9,10]. Among them, solar cells are considered the most attractive ways to convert solar energy into electricity directly [11,12,13,14,15,16,17]. Nowadays, the commercialized photovoltaic devices are silicon solar cells, showing high power conversion efficiencies (PCEs) and superb operational stability [18,19]. Nevertheless, silicon solar cells suffer from high production costs, complex fabrication processes and toxic by-products [20]. Thus, it is necessary to develop other alternative solar cells with low cost, high PCE and environmental friendliness [21,22,23]. Perovskite solar cells (PSCs) as one of the third-generation solar cells have received particular interest because of the large absorption coefficient of organic-inorganic halide perovskites, simple preparation process and relatively high PCEs [24,25,26]. The PCEs of PSCs have been boosted from 3.8% to 25.7% since their invention in 2009, which is comparable to that of silicon solar cells [27,28]. Nevertheless, the widespread applications of PSCs are still limited by several crucial factors such as inferior long-term stability of organic-inorganic halide perovskites, limited choices of key cell components as well as high costs of hole-transporting materials and metal electrodes, etc. [29,30,31].

**Figure 1:**
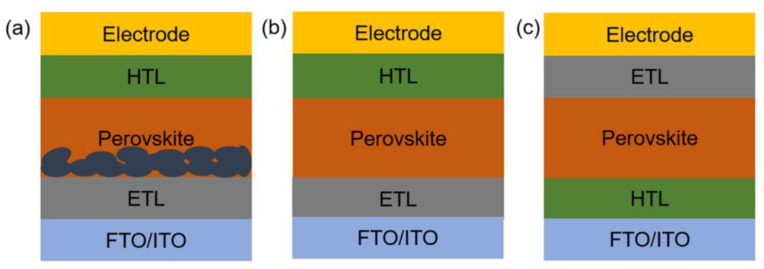
Schematic diagrams of PSCs with different configurations. (**a**) mesoporous n-i-p structure; (**b**) planar n-i-p structure; (**c**) planar p-i-n structure.

The PSCs can be categorized into mesoporous and planar configurations based on the structure, as well as normal (n-i-p) and inverted (p-i-n) configurations based on the charge transfer mechanism, as displayed in Figure 1. Typically, PSCs are composed of conducting glass such as fluorine tin oxide (FTO) or indium tin oxide (ITO), electron-transporting layer (ETL), perovskite absorption layer, hole-transporting layer (HTL) and the metal electrode (Figure 1). As for the working mechanism of PSCs, photons are absorbed on the perovskite absorption layer under sunlight irradiation to create electron-hole pair, which can be effectively separated at room temperature owing to the low exciton binding energy of halide perovskites and then transferred through ETL and HTL to the electrodes, respectively [32,33,34]. However, unavoidable recombination and accumulation of charge carriers are often experienced in PSCs under actual working conditions, leading to inferior PCEs and stability [35,36]. Numerous efforts have been devoted to boosting the PCEs and durability of PSCs during the past 5 years, and several effective strategies have been proposed, including material design for ETL, perovskite layer and HTL, additive engineering, interface control, solvent engineering and cell configuration design [26,29,30,36,37,38,39].

In addition to the inferior stability of organic-inorganic halide perovskites under humid and high-temperature conditions, the instability issue hindering the practical applications of PSCs is also mainly caused by the poor heat/moisture tolerance and molecular structural instability of state-of-the-art organic HTLs [40,41,42]. In PSCs, the HTL is a p-type semiconductor to transport holes from the perovskite film to the metal electrode. In addition, HTL also functions as a barrier to block the electron transfer from perovskite film and to avoid the direct contact between the perovskite layer and the electrode in order to reduce carrier recombination [33,43,44]. Therefore, an excellent HTL material should have high hole extraction/mobility and suitable highest occupied molecular orbit (HOMO)/valence band maximum (VBM) with the HOMO of the perovskite layer to achieve a proper energy level alignment [45,46,47]. At present, the most commonly used HTL is 2,2′,7,7′-Tetrakis (N,N-di-p-methoxyphenyl-amine)-9,9′-spirobifluorene (Spiro-OMeTAD), showing suitable energy level alignment with perovskite layer and the metal electrode [48,49]. However, Spiro-OMeTAD must be modified by lithium salts or 4-tert-butylpyridine to exhibit high hole mobility, which unfortunately increases the hygroabsorption capability, leading to inferior moisture stability [50,51]. In addition, most of the widely used organic HTLs for PSCs, including poly(3,4-ethylenedioxythiophene) polystyrene sulfonate (PEDOT:PSS), poly(triarylamine) (PTAA), suffer from the same drawback of poor thermal stability [52,53].

**Figure 2:**
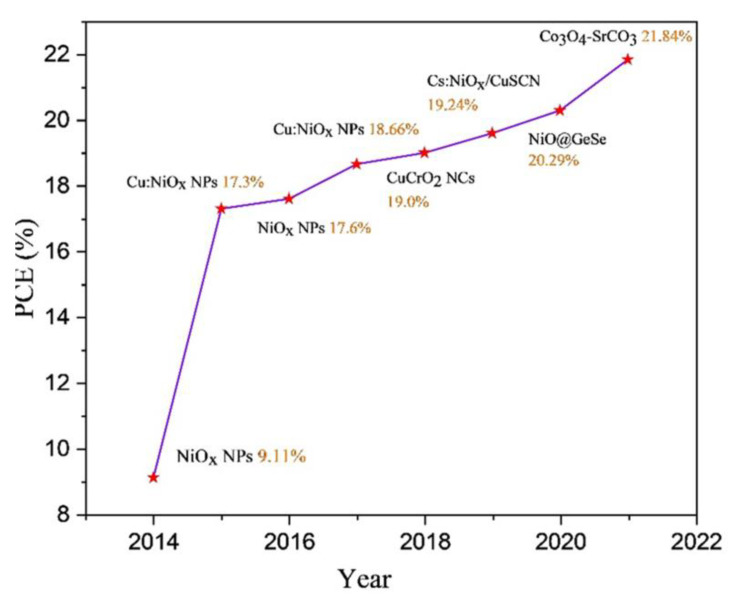
The PCEs evolution of PSCs with nanostructured inorganic HTLs [60,61,62,63,64,65,66,67].

Thus, inorganic HTLs with high intrinsic structural and thermal stability are regarded as highly promising alternatives to replace organic counterparts for stable PSCs [54,55]. Particularly, the nanostructure construction is also reported to effectively enhance the hole transfer capability of inorganic HTLs [56,57]. For instance, a number of nanostructured inorganic HTLs have been designed and developed for PSCs such as metal oxide/sulfide-based nanostructures, including NiO_x_ nanoparticles (NPs), CuIn_0.5_Ga_0.5_S_2_ NPs and Cu_2_ZnSnS_4_ NPs, CuSCN NPs, CuGaO_2_-CuSCN nanocomposite, etc., due to the relatively superb chemical stability, high hole mobility, tunable band gap and low cost [37,57,58,59]. Figure 2 shows the PCEs evolution of PSCs with nanostructured inorganic HTLs since the first report in 2014. The PCEs of nanostructured inorganic HTL-based PSCs rapidly increased from 9.11% to 21.84% during the past 8 years [60,61,62,63,64,65,66,67]. Nanostructured inorganic HTLs have been widely used in both normal and inverted PSCs. Particularly, nanostructured inorganic HTLs have attracted increasing interest in inverted PSCs due to the excellent light transmission capability and superior surface morphology, which is beneficial for the further deposition of high-quality perovskite films [68,69]. However, the PCEs of nanostructured inorganic HTL-based PSCs are still far away from the cells with organic counterparts, which requires further investigations. In this paper, the recent advances in the design and fabrication of nanostructured inorganic HTLs for PSCs are summarized and discussed by highlighting the prerequisites of HTLs and the superiority of nanostructured inorganic HTLs over organic counterparts. Furthermore, several relevant strategies to boost the performance of inorganic HTLs are presented, including fabrication route design, functional/selectively doping, morphology control, nanocomposite construction, crystal structure regulation and bilayer HTL construction. At last, the remaining problems and future research directions about nanostructured inorganic HTL-based PSCs are also discussed. This review aims to present some insights for the future development and fabrication of high-efficiency and durable inorganic HTL-based PSCs.

## 2. Current Research Status of HTLs in PSCs

### 2.1. The Crucial Roles of HTLs in PSCs

The HTLs play an important role in PSCs to effectively extract holes from the perovskite film and to transport holes to the metal electrode in normal PSCs [70,71]. In addition, HTLs also function as a barrier to prevent the direct contact of the perovskite film and metal electrode, reducing the potential recombination of electrons and holes in normal PSCs [72,73]. Particularly, the HTL also can significantly affect the grain sizes and grain boundary amount of the perovskite layer in inverted PSCs, which strongly influences the PCEs and long-term stability of PSCs [74,75]. Therefore, an ideal HTL should have the following prerequisites [41,57,76,77]. First, HTLs should have high hole mobility to transfer photogenerated holes to the electrode, which is beneficial for the achievement of a high fill factor (FF). It has been reported that when the hole mobility of HTLs was enhanced from 10^−6^ to 10^−4^ cm^2^ V^−1^ s^−1^, FF values of corresponding PSCs can be boosted from 0.22 to 0.80 [78]. Second, HTLs should display superior hydrophobicity, (photo)chemical and thermal stability to enhance the durability of PSCs. Third, the low cost of HTLs is also required to promote large-scale production. Forth, a suitable energy level alignment between HTLs and perovskite film is required to reduce the carrier recombination and the hole accumulation at the perovskite film/HTL interface. Fifth, the high film quality of HTLs is also required to improve the coverage of the perovskite film and to promote the deposition of high-quality perovskite film in the normal and inverted PSCs, respectively. The ideal HTL for PSCs should have no interfacial reaction with other functional layers, such as the perovskite film and the metal electrode [79,80]. The chemical corrosion of metal electrodes on the organic HTLs can be inhibited in inorganic HTL-based PSCs due to the more stable interface between inorganic HTL and the metal electrode [81,82]. Until now, the PCEs of inorganic HTL-based PSCs are still lower than those of organic HTL-based PSCs mainly due to the detrimental interfacial reaction between inorganic HTLs and perovskite film, which reduced the hole extraction capability and increased the carrier recombination rate at interfaces, leading to serious open circuit voltages (V_OC_) loss and accelerated decomposition of perovskite film [83,84]. For example, Ni^3+^ cations in NiO_x_ HTL can react with the A-site cationic salts in the perovskite precursors, which reduces the amount of Ni^3+^ cations in NiO_x_, leading to lower conductivity of NiO_x_ HTL and reduced PCEs of PSCs [85]. In order to reduce the interfacial reaction between the HTL and the perovskite film, Zhang et al. introduced a 2-methyl-1-aziridinepropionate (SaC-100) layer between NiO_x_ and perovskite film to achieve a physical separation [86]. The introduction of the SaC-100 layer not only effectively inhibited the redox reactions at the HTL/perovskite film interface but also increased the amount of Ni^3+^ cations in NiO_x_ HTL, which efficiently reduced the interfacial defect amount and improved the conductivity of NiO_x_ HTL. Consequently, SaC-100 modified cells exhibited a much higher PCE of 20.21% than that of the unmodified PSC (17.54%). In addition, self-assembled monolayers (SAMs) are also widely employed to reduce interfacial reactions in PSCs due to the ultra-thin, uniform and high-quality film of SAMs, which can effectively passivate interfacial defects, improve interface contact and reduce the V_OC_ loss in PSCs [87]. For instance, Mangalam et al. modified the interface between NiO_x_ HTL and perovskite film by using 4-bromophenylphosphonic acid-based SAMs, which increased the V_OC_ from 0.978 V to 1.029 V, effectively improving the PCEs of PSCs [88]. Up to now, organic HTLs based on polymer small molecules, including PEDOT:PSS, Spiro-OMeTAD, etc., have been widely used in PSCs, while inorganic HTLs such as NiO_x_, CoO, ZnCo_2_O_4_ and Fe_3_O_4_ have received increasing attention during the past 5 years [39,57,89,90,91].

### 2.2. Advantages and Disadvantages of Organic HTLs

At present, the most commonly used HTL in PSCs is organic Spiro-OMeTAD, exhibiting relatively high PCEs. Nevertheless, Spiro-OMeTAD suffered from several drawbacks such as high cost, complex synthesis process, poor stability, etc. Furthermore, the PSC with pristine Spiro-OMeTAD delivered a low PCE of 9.7% because of the inferior hole mobility and conductivity induced by the disordered molecule structure of pristine Spiro-OMeTAD [92]. The hole mobility of Spiro-OMeTAD was remarkably enhanced by adding lithium bis(trifluoromethanesulfonyl)imide, 4-tert-butylpyridine and cobalt(III) tris(bis(trifluoromethyl-sulfonyl)imide) to achieve a high PCE of 19.7% [93]. However, the introduction of these additives also brings some unstable factors, which reduce the stability of PSCs [92,94]. For instance, the addition of lithium salts led to inferior moisture stability of PSCs due to the hygroscopic nature, while a detrimental reaction between 4-tert-butylpyridine and halide perovskite accelerated the decomposition of perovskite film during operation [95]. Very recently, Li et al. employed polymethyl methacrylate (PMMA) to modify Spiro-OMeTAD to boost the PCE and moisture stability of PSCs by reducing the interaction between Spiro-OMeTAD/perovskite layers and water [96]. As a result, the cell with PMMA-modified Spiro-OMeTAD produced an attractive PCE of 21.08%, which was much better than the unmodified counterparts. Furthermore, the PCE of the cell with PMMA-modified Spiro-OMeTAD maintained 77% of the initial value after storing in the air with relative humidity (RH) of 40% for 80 days, while the pristine cell deteriorated to 47% of the initial PCE under the same conditions. Besides additive engineering, some researchers have also modified Spiro-OMeTAD by regulating the functional groups in the molecule structure [97,98]. For instance, Jeon et al. tailored the position of methoxy group (para, ortho and meta) in the benzene ring connected with N atom in Spiro-OMeTAD, which significantly affected the electrical and optical properties [97]. It was found that the methoxy group located in the ortho position in Spiro-OMeTAD increased the lowest occupied molecular orbital (LUMO) value of pristine Spiro-OMeTAD while the methoxy group at meta and para positions reduced the LUMO value of pristine Spiro-OMeTAD. Consequently, the cell with ortho-Spiro-OMeTAD exhibited a higher PCE of 16.7% than those of pristine Spiro-OMeTAD (15.2%), meta-Spiro-OMeTAD (13.9%) and para-Spiro-OMeTAD (14.9%) due to the promoted electron blocking and enhanced hole transfer. PEDOT:PSS is a commonly used organic HTL in inverted PSCs due to its superior optical transparency and high conductivity [99,100]. It has been reported that a superb PCE of 20.1% was obtained by using such PEDOT:PSS HTL in inverted PSCs [101]. Nevertheless, the hydrophilic and acidic nature of PEDOT:PSS led to inferior durability of PSCs [102]. Moreover, the V_OC_ of corresponding cells are always less than 0.95 V, which was caused by the energy level mismatch between perovskite film and PEDOT:PSS layer [103]. In summary, although organic HTLs displayed superior hole mobility, the inherent instability under high-temperature and humid conditions remarkably limited further applications in PSCs. Consequently, it is essential to seek intrinsic durable alternative HTLs (e.g., inorganic materials) to achieve high-efficiency and durable PSCs [104,105,106].

### 2.3. The Superiority of Inorganic HTLs and the Importance of Nanostructure Construction

Recently, inorganic materials have emerged as new-generation HTLs to replace organic counterparts due to several distinct advantages such as superior hole collection/migration capability, high conductivity, stability and light transmittance, easy adjustment of energy levels, low cost, simple fabrication, etc. [56,57,58,69]. Until now, several inorganic materials have been utilized as HTLs for PSCs, including NiO_x_, CuO_x_, CuS, MoS_2_, VO_x_, WO_3_, CuSCN, etc. [41,55,56,57,58,59]. Since the first investigation of inorganic HTLs for PSCs, numerous efforts have been devoted to boosting the PCEs of inorganic HTL-based PSCs [56,64,68,107]. Fortunately, the obtained PCEs of inorganic HTL-based PSCs are now comparable to those of their organic counterparts. In 2014, Wang et al. firstly employed the spin coating method to fabricate NiO_x_ HTL in mesoporous PSCs to obtain a PCE of 9.51% [108]. In addition to the development of fabrication methods, interface engineering and functional doping have also been employed to boost the PCEs of inorganic HTL-based PSCs [109,110]. For instance, Yip et al. modified NiO_x_/MAPbI_3_ interface with diethanolamine (DEA) to improve the charge extraction capability and to reduce the charge recombination rate, and a high PCE of 18.1% was obtained [109]. As for the functional doping, Xiang et al. co-doped NiO_x_ by lithium (Li^+^) and magnesium (Mg^2+^) as HTLs for PSCs [110]. The cell with such co-doped NiO_x_ exhibited a superior PCE of >20%, which was assigned to the improved conductivity and more proper energy level alignment induced by Li^+^ and Mg^2+^ doping, respectively. Until now, the solution-based routes are the most widely used methods to fabricate inorganic HTLs for PSCs because of their excellent universality and cost-effectiveness.

Nevertheless, the bulk inorganic HTLs suffered from large particle sizes, which were very difficult to be well dispersed in the solution. As a result, many pinholes and cracks were formed in the solution-processed bulk inorganic HTLs, leading to increased carrier recombination and an inferior coverage on the perovskite layer, which strongly affected the PCEs and stability of corresponding PSCs. On this basis, nanostructure construction is introduced to develop inorganic HTLs with nanoparticles and special morphologies to enhance the PCEs of PSCs. Several promising nanostructured inorganic HTLs such as NiO_x_ NPs, CoO NPs, Fe_3_O_4_ NPs, Cr/CuGaO_2_-CC/NiO_x_ nanocomposites, NiCo_2_O_4_ NWs, etc., have been designed and developed to boost the PCEs of PSCs due to smaller grain sizes and better dispersion in solution than the bulk counterparts, leading to more compact and flatter hole-transporting films [57,90,91,111,112,113]. In addition, nanostructured inorganic HTLs not only effectively reduced the reflection loss caused by light scattering and improved the light capturing efficiency but also increased the interfacial contact area between HTL and perovskite film, which significantly improved the carrier extraction rate and suppressed the charge recombination. In this review, the recent advances in the design and development of nanostructured inorganic HTLs for PSCs are summarized and discussed. Several strategies, including fabrication route design, functional/selectively doping, morphology control, nanocomposite construction, crystal regulation and bilayer HTL design, are also proposed, which are discussed in the following section.

## 3. Advances in the Nanostructured Inorganic HTLs for PSCs

### 3.1. Fabrication Route Design

For nanostructured inorganic HTL-based PSCs, the film quality and surface morphology of nanostructured inorganic HTLs play a vital role in achieving high PCEs and stability of PSCs, which are closely related to the fabrication methods of HTLs. Therefore, the design and optimization of the fabrication routes for nanostructured inorganic HTL-based PSCs have received increasing attention. At present, NiO_x_ is one of the most investigated nanostructured inorganic HTLs in PSCs because of its high light transmittance, good chemical stability, wide band gap (3.6–4.0 eV) and proper energy level alignment with perovskite film [65,114]. Furthermore, it has also been reported that the fabrication route for NiO_x_ HTL strongly influences the performance of corresponding PSCs [115]. Generally, nanostructured NiO_x_ HTLs are fabricated by spray pyrolysis, which suffers from high-temperature annealing, leading to inferior cell performance [65,115]. Therefore, numerous efforts have been carried out for the design of fabrication routes for nanostructured NiO_x_ HTLs such as sol-gel, pulsed laser deposition (PLD), solvothermal, etc. [114,115]. For instance, in 2014, Zhu et al. prepared NiO NCs-based HTLs for MAPbI_3_-based PSCs by sol-gel route [60]. It was found that polyhedral NiO nano single crystals were formed on the NiO film surface, enabling an intimate and large interface area connection with MAPbI_3_ film, which significantly improved the cell efficiency. More specifically, the uniform NiO NCs also promoted the lead iodide (PbI_2_) deposition in the two-step perovskite film fabrication processes, while large MAPbI_3_ grains were formed to construct a highly textured MAPbI_3_/Methyl [6,6]-phenyl-C_61_-butyrate (PCBM) interface with the help of uniform NiO NCs surface, which remarkably increased the electron-hole diffusion length. In addition, NiO NCs also acted as effective light scattering centers to enhance the sunlight absorption capability of PSCs. Based on the above merits induced by NiO NCs as HTLs, the inverted PSC delivered a PCE of 9.11%. Afterward, Park et al. employed PLD to fabricate ordered nanostructured NiO film, showing superior optical transparency and optimized nanostructure by tailoring the deposition time and oxygen partial pressures [61]. Inverted PSCs with planar heterojunction of MAPbI_3_/PCBM and 30 nm-thick NiO HTL exhibited a superior PCE of 17.3%, which was assigned to the remarkably suppressed recombination rate and improved extraction capability of charge carriers as well as the high light transmittance of ~95% of such optimized NiO HTL. In 2018, Tang et al. employed a solvothermal route to synthesize high-crystallinity NiO NCs by using olamine ligands to tailor the nucleation and growth processes of NiO NCs to obtain superb colloidal stability in toluene [116]. The PSC with 55 nm thick NiO NCs-based HTL generated a superior PCE of 15.47% to the cell with conventional organic PEDOT:PSS HTL (11.42%) due to the much-improved light transmittance. Furthermore, the NiO NCs-based unencapsulated cell maintained 65% of the primary PCE after 96 h’s storage in ambient conditions at 25 °C, while no PCE was retained for PEDOT:PSS-based counterparts under the same conditions.

**Figure 3:**
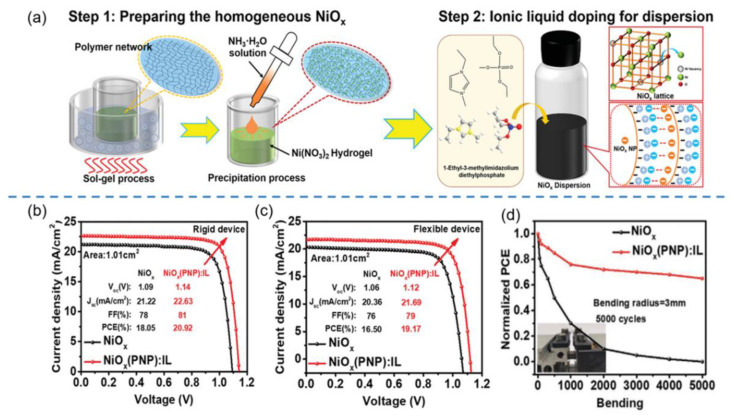
(**a**) Schematic diagrams of highly dispersed NiO_x_ colloidal solutions prepared by PNP-based sol-gel method; (**b**) J–V curves of the rigid PSCs with NiO_x_ and NiO_x_-PNP-EMDP IL as HTLs; (**c**) J–V curves of flexible PSCs with NiO_x_ and NiO_x_-PNP-EMDP IL as HTLs; (**d**) the PCE stability of flexible PSCs with NiO_x_ and NiO_x_-PNP-EMDP IL HTLs as a function of the number of bending cycles. Reproduced from [118], with permission from the Advanced Functional Materials, 2021.

In addition to the design of new fabrication methods for nanostructured NiO_x_ HTLs, the modification of the widely used sol-gel process has also been utilized to fabricate high-performance NiO_x_ HTL-based PSCs. For instance, Tong et al. developed a new soft base precipitation method to fabricate NiO_x_ HTLs by replacing NaOH with ammonia in the sol-gel process [117]. The influences of ammonia contents (from 1 to 6, molar ratio) on the crystallization behavior and particle sizes of NiO_x_ NPs were systematically investigated. After optimizing the ammonia content (molar ratio of 1:3), the cell with a configuration of ITO/NiO_x_ NPs/FASnI_3_/PCBM/Bathocuproine (BCP)/Ag generated a superior PCE of ~8% due to the improved light transmittance and film quality of NiO_x_ HTL. In another work, Zhang et al. synthesized highly monodispersed NiO_x_ NPs by sol-gel method based on polymer network precipitation (PNP), as depicted in Figure 3a [118]. During the dispersion of NiO_x_ NPs in solution, 1-ethyl-3-methylimidazolium diethyl phosphate ionic liquid (EMDP IL) was added as the surfactant to obtain a highly dispersed NiO_x_ colloid solution. As a result, PSCs with such NiO_x_-PNP-EMDP IL HTL delivered high PCEs of 20.92% and 19.17% on rigid and flexible substrates, as displayed in Figure 3b,c, which were much higher than the cells with pristine NiO_x_ HTL (18.05% and 16.50%), respectively. Furthermore, the PCE stability of flexible PSCs was also tested, with results shown in Figure 3d. NiO_x_-PNP-EMDP IL HTL-based device retained 60% of the initial PCE after 5000 bending cycles, while no PCE was retained for the cell with pristine NiO_x_ HTL under the same conditions. Zhang et al.’s study provides a new strategy for the fabrication of flexible and large-area PSC with high-quality NiO_x_ NPs as HTLs.

**Figure 4:**
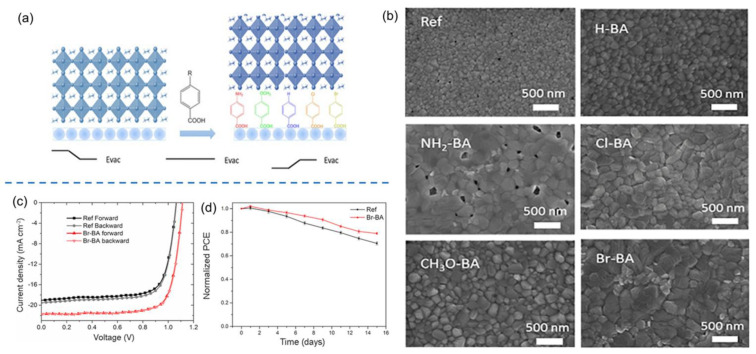
(**a**) Schematic diagram of NiO_x_/MAPbI_3_ interface as modified by various BA-based SAMs; (**b**) top-view SEM images of perovskite films deposited on the NiO_x_ HTLs without and with modification by various BA-based SAMs; (**c**) J–V curves of PSCs with the pristine NiO_x_ and Br-BA modified NiO_x_ as HTLs under forward and reverse scan directions; (**d**) stability tests of PSCs with the pristine NiO_x_ and Br-BA modified NiO_x_ as HTLs in ambient conditions with an RH of 30%. Reproduced from [120], with permission from the ChemSusChem, 2017.

Besides the above-mentioned fabrication methods, room temperature solution-processing is also regarded as a facile and effective strategy to design and develop nanostructured NiO_x_ HTLs. For instance, Zhang et al. used a room-temperature solution treatment to fabricate defect-free nanostructured NiO_x_ HTLs [62]. The flexible and rigid PSCs with such NiO_x_ film exhibited PCEs of 14.53% and 17.6%, respectively. Chen et al. systematically compared the performance of NiO_x_ NPs obtained by room-temperature (L-NiO_x_) and high-temperature (H-NiO_x_) treatments for application as HTLs for mixed Sn-Pb perovskite-based PSCs [119]. It was found that the L-NiO_x_ film displayed a more compact/smoother morphology and better energy level matching with Sn-Pb perovskite layer than those of H-NiO_x_. Consequently, the cell with L-NiO_x_ HTL produced a much higher PCE of 18.77% than the H-NiO_x_-based device (15.85%) due to the enhanced film quality of HTL and promoted hole extraction from the perovskite layer to HTL. Furthermore, the PCE of the cell with L-NiO_x_ HTL retained 96% of the initial value after storing in the N_2_ atmosphere for 50 days, which was assigned to the high-quality perovskite and HTL films, low interfacial defect density and inhibited self-oxidation of Sn^2+^ in Sn-Pb perovskites. In another work, Wang et al. various kinds of benzoic acid (BA)-based SAMs to passivate NiO_x_ NPs-based HTLs at HTL/perovskite interface by room temperature treatment as depicted in Figure 4a [120]. Based on the top-view SEM images of perovskite films deposited on NiO_x_ NPs-based HTLs after modification by BA-based SAMs in Figure 4b, 4-bromobenzoic acid (Br-BA) played the best role in the surface defect passivation of perovskite film as evidenced by the largest grain sizes and pinhole-free morphology. As depicted in Figure 4c, the cell with Br-BA modified NiO**_x_** HTL displayed a superior PCE of 18.4% with negligible hysteresis effect, which was much higher than that of the cell with pristine NiO**_x_** NPs as HTL (15.3%) owing to the better crystallinity, suppressed redox reactions between interfaces, reduced amount of grain boundaries and larger grain sizes of perovskite layer after the modification of Br-BA SAMs. Moreover, the cell with Br-BA modified NiO_x_ HTL showed better stability than the unmodified PSCs after storing in ambient conditions with an RH of 30% for 15 days, as depicted in Figure 4d, which was mainly due to the reduced trap-assisted recombination, increased energy level alignment between NiO_x_ HTL and perovskite film, the tailored surface wettability of HTL and superior quality of perovskite film.

### 3.2. Functional/Selectively Doping

Although the design of advanced fabrication methods can effectively reduce the aggregation of NiO_x_ NPs and/or its dispersion in solution, the low intrinsic conductivity of pristine NiO_x_ is still much lower than the organic counterparts, which was assigned to the large ionization energy of Ni vacancies [3,77]. In order to tackle this problem, functional/selectively doping (e.g., Co^2+^, Ni^3+^, Fe^3+^ and Cu^+^) has been reported as a facile and effective strategy to boost the conductivity of nanostructured NiO_x_ as HTLs for PSCs [63,65,121,122,123]. For instance, Kaneko et al. reported the Co doping into NiO_x_ NPs prepared by chemical precipitation at different Co doping concentrations (0–10 mol.%) [121]. It was found that 0.5 mol.% Co-NiO_x_ NPs exhibited the best surface morphology while higher light transmittance was achieved at the Co doping amount of 0.5–2 mol.%. In addition, the conductivity of pristine NiO_x_ was remarkably increased from 3.83 to 6.20 × 10^−^^6^ S cm^−^^1^ at a Co doping concentration of 5 mol.% due to the increased amount of high-valence Ni^3+^ cations while excessive Co doping amount (10 mol.%) reduced the conductivity of NiO_x_. A maximum PCE of 14.5% was achieved by the Co-NiO_x_-based PSC at a Co doping amount of 1 mol.%, which may be assigned to the balance between surface morphology, light transmittance and conductivity of Co-NiO_x_ HTL. Similar results were also reported by Chandrasekhar et al. about the development of Fe doped NiO_x_ HTLs at different concentrations of 0–2 mol.% [122]. The cell with a configuration of ITO/Fe-NiO_x_/MAPbI_3_/PCBM/BCP/Ag at Fe doping amount of 1 mol.% exhibited the highest PCE of 17.59%, 14% higher than that of the cell with NiO_x_ HTL, which was mainly attributed to the increased conductivity and work function of NiO_x_ induced by Fe doping at a proper concentration.

**Figure 5:**
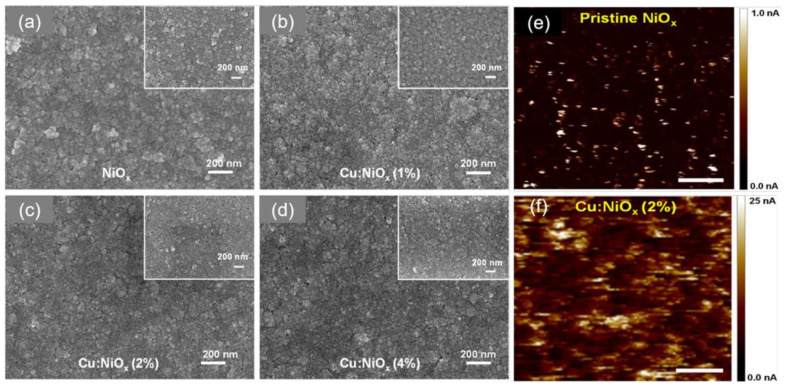
SEM images of (**a**) NiO_x_, (**b**) 1 mol.% Cu doped NiO_x_, (**c**) 2 mol.% Cu doped NiO_x_ and (**d**) 4 mol.% Cu doped films deposited on flexible substrates (SEM images of corresponding films on rigid substrates are shown inset); AFM images of (**e**) NiO_x_ and (**f**) 2 mol.% Cu doped NiO_x_ films. Reproduced from [63], with permission from the ACS Applied Materials & Interfaces, 2017.

**Figure 6:**
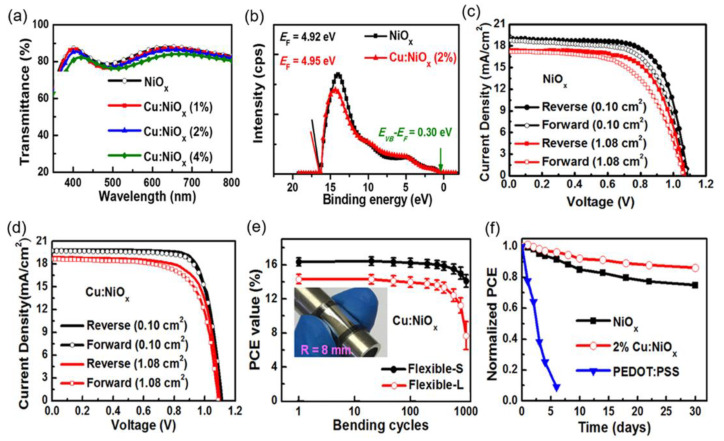
(**a**) Optical transmission spectroscopy of NiO_x_ and various Cu doped NiO_x_ films deposited on the quartz glass; (**b**) Ultraviolet photoelectron spectroscopy (UPS) spectra of NiO_x_ and 2 mol.% Cu doped NiO_x_ films; J–V curves of flexible PSCs with (**c**) pristine NiO_x_ and (**d**) 2 mol.% Cu doped NiO_x_ as HTLs under forward and reverse scan directions; (**e**) the PCE stability of flexible PSCs with 2 mol.% Cu doped NiO_x_ HTL as a function of the number of bending cycles; (**f**) ambient stability of PSCs with NiO_x_, 2 mol.% Cu doped NiO_x_ and PEDOT:PSS as HTLs. Reproduced from [63], with permission from the ACS Applied Materials & Interfaces, 2017.

In another work, He et al. systematically investigated the influences of Cu doping amount on the film quality, light transmittance and hole mobility of NiO_x_ NPs as HTLs for PSCs [63]. As depicted in Figure 5 and Figure 6a,b, the optimized 2 mol.% Cu doped NiO_x_ HTL displayed the best surface morphology and light transmittance (>80%) as well as increased work function, which was beneficial for the hole transporting between perovskite film and the HTL. Furthermore, the 2 mol.% Cu doping also significantly improved the hole mobility of NiO_x_ from 3.05 × 10^−^^3^ to 1.05 × 10^−^^2^ cm^2^ V^−^^1^ s^−^^1^. Consequently, the 2 mol.% Cu doped NiO_x_-based PSCs with active areas of 0.1 and 1.08 cm^2^ exhibited higher PCEs of 18.66% and 15.10% than those of NiO_x_ NPs-based PSCs (15.47% and 11.51%, respectively) as depicted in Figure 6c,d. In addition, the 2 mol.% Cu doped NiO_x_-based flexible cells also displayed excellent PCE stability for 500 bending cycles (Figure 6e). As depicted in Figure 6f, it is found that 2 mol.% Cu doped NiO_x_-based PSC maintained 86% of the initial PCE after 30 days’ storage under ambient conditions with an RH of 40% at 25 °C, while the undoped NiO_x_-based PSC only retained 75% of the primary PCE under the same conditions. More importantly, no PCE was retained for the PEDOT:PSS-based PSC after 7 days’ storage as depicted in Figure 6f, highlighting the crucial roles of Cu-doped NiO_x_ HTL in boosting the cell stability. Similarly, Chen et al. prepared HTL films by spin-coating Cu-doped NiO NPs-based ink at room temperature to achieve a high PCE of > 20% without any post-treatment, which was mainly due to the co-existence of Cu^+^ and Cu^2+^ cations after Cu doping in NiO to improve the hole extraction [123].

**Figure 7:**
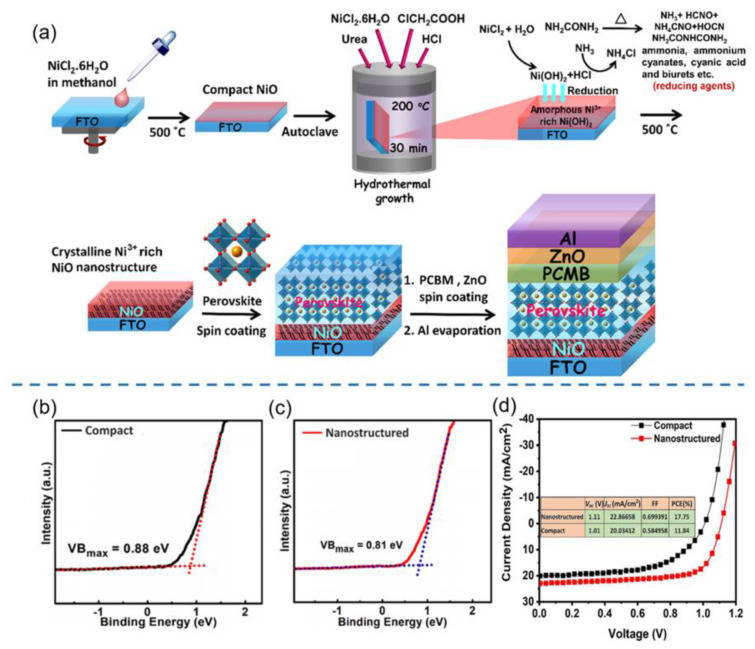
(**a**) Schematic diagrams of the preparation process of Ni^3+^ doped NiO by hydrothermal method and corresponding PSCs; the UPS spectra of (**b**) pristine NiO film and (**c**) Ni^3+^-NiO NWs film; (**d**) J–V curves of PSCs with pristine NiO and Ni^3+^-NiO NWs as HTLs. Reproduced from [125], with permission from the ACS Applied Materials & Interfaces, 2020.

Besides monovalent and divalent cations, the doping of trivalent cations can also be used to remarkably boost the conductivity of NiO_x_, especially for the trivalent lanthanide cations with partially filled 4f orbitals and empty 5d orbitals [124]. For instance, Bao et al. synthesized samarium-doped nickel oxide (Sm:NiO_x_) NPs by a chemical precipitation route and deposited them as HTLs for PSCs [124]. It was found that the Sm^3+^ doping suppressed the formation energy of Ni vacancies and increased the amount of Ni vacancies to increase the hole density. Thus, the conductivity of NiO_x_ was improved remarkably by Sm^3+^ doping while the work function was enlarged in Sm:NiO_x_ HTL, which promoted the hole extraction and reduced the charge recombination at the HTL/perovskite interface. Thus, the cell with Sm:NiO_x_ HTL exhibited a superior PCE of 19.16% to that of the NiO_x_-based cell (17.23%). After optimizing the compositions of perovskite film, high PCEs of 20.71% and 17.95% were achieved for rigid and flexible PSCs based on Sm:NiO_x_ HTL, respectively. Particularly, Sm:NiO_x_ also showed promises for application in large-area PSCs. Sm:NiO_x_-based PSC with active areas of 1 and 16 cm^2^ produced attractive PCEs of 18.51% and 15.27%, respectively, which were among the highest PCEs of NiO_x_-based PSCs with large areas. Bao et al.’s work emphasizes a new doping strategy for the development of high-quality NiO_x_ HTLs in conjunction with revealing the doping effect in depth, which can provide some guidelines for the fabrication of high-efficiency and flexible PSCs in the future. As mentioned above, high-valence Ni^3+^ cations are beneficial for the achievement of the high conductivity of NiO HTL [125]. Thakur et al. synthesized Ni^3+^ doped NiO NWs (Ni^3+^-NiO NWs) by hydrothermal method, as depicted in Figure 7a [125]. Firstly, the precursor solution composed of urea, chloroacetic acid, deionized water and hydrochloric acid is prepared and various concentrations of NiCl_2_·6H_2_O (1, 3 and 5 mM) are then dissolved in the precursor solution. Then, FTO/NiO was immersed into the precursor solution, which was further hydrothermally treated at 200 °C for 0.5 h to realize the hydrothermal growth of NPs. NiCl_2_ was hydrolyzed into Ni^3+^-rich Ni (OH)_2_ by reducing chemicals including ammonium cyanate, ammonia and cyanic acid decomposed by urea, which was further annealed at 500 °C to obtain Ni^3+^-NiO NWs. After optimizing the NiCl_2_·6H_2_O concentration (1 mM), Ni^3+^-NiO NWs HTL displayed the best surface morphology. In addition, the VBM value of Ni^3+^-NiO NWs (0.83 eV) was smaller than that of pristine NiO film (0.88 eV), as depicted in Figure 7b,c due to the porous structure of Ni^3+^-NiO NWs with Ni atoms exposed at the edge, which effectively promoted the hole transport. As a result, the cell with Ni^3+^-NiO NWs HTL produced an excellent PCE of 17.75%, which was much better than the pristine NiO-based cell (11.5%), as depicted in Figure 7d.

In addition to NiO_x_, Sb_2_S_3_ is also regarded as a promising hole-transporting material for PSCs because of the high carrier density, superior hole mobility, wide band gap, etc. For instance, Mohamadkhan et al. synthesized Sb_2_S_3_ and Cu doped Sb_2_S_3_ (Cu_3_SbS_4_) NCs by an oil amine-assisted heating method [126]. Cu_3_SbS_4_ NCs-based PSCs exhibited a higher PCE of 13.0% that of the Sb_2_S_3_-based cell (8.2%) due to the increased conductivity and carrier density, improved charge transport/extraction capability of Cu_3_SbS_4_ NCs induced by Cu doping. Azam et al. systematically investigated the influences of Ga doping amount on the performance of Cu(In_1−x_Ga_x_)S_2_ (CIGS_x_, x = 0–0.75) NPs as inorganic HTLs for PSCs [127]. It was found that with the increase in Ga doping concentration, CIGS displayed a decreased valence band edge and an increased conduction band edge, which improved the electron-blocking capability and suppressed the carrier recombination rate. The cell with optimized CIGS_0.5_ HTL showed an attractive PCE of 15.6%, which was much better than the cell with pristine CuInS_2_ (10.85%). In addition, the cell with CIGS_0.5_ HTL retained 70% of the primary PCE after aging in the dark conditions with an RH of 50% for 90 days, while the cell with Spiro-OMeTAD HTL only retained <50% of the original PCE under the same conditions.

### 3.3. Morphology Control

It is well known that the morphologies of inorganic HTLs as substrates for the deposition of perovskite film have significant impacts on the quality and grain sizes of perovskite film, which further influences the photovoltaic performance of PSCs. On this basis, some advanced morphologies of inorganic HTLs, including NPs, NWs, nanorods (NRs) and nanosheets (NSs), have been synthesized by controlling the fabrication parameters and designing new preparation methods. For instance, Liu et al. prepared NiO NSs by a solution extraction method at room temperature for the application as HTLs for carbon electrode-based PSCs [128]. It was found that NiO NSs-based PSC produced a high PCE of 14.2%, which was 15% higher than that of NiO NPs-based counterparts due to the improved hole collection efficiency, longer charge lifetime and better contact with perovskite film. Remya et al. prepared WO_3_ nanostructures with different morphologies, including WO_3_ NPs and WO_3_ NSs, by a solution treatment route, which were employed as alternatives to organic HTLs in PSCs [129]. It was found that the cell with WO_3_ NSs HTL exhibited a higher PCE of 7.76% than that of WO_3_ NPs-based counterparts (6.83%) due to the enhanced hole collection efficiency and hole mobility induced by the NSs-like morphology.

**Figure 8:**
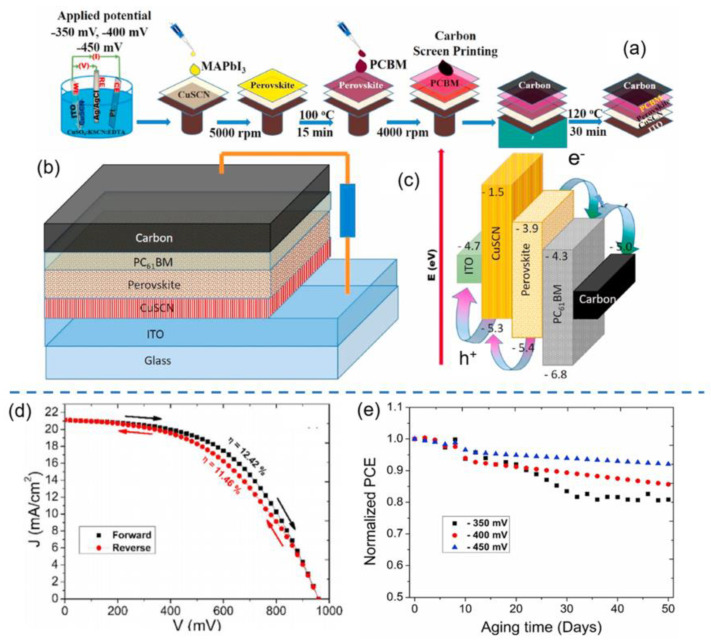
(**a**) Schematic diagrams of the preparation processes of CuSCN nanostructures by electrodeposition method and corresponding PSCs; (**b**) device configuration and (**c**) energy level diagrams of CuSCN HTL-based PSC; (**d**) J–V curves of PSCs based on CuSCN NRs HTLs; (**e**) the ambient stability of PSCs based on CuSCN HTLs with different morphologies. Reproduced from [130], with permission from the Solar Energy Materials and Solar Cells, 2021.

Recently, Ramachandran et al. employed a simple electrochemical deposition method to fabricate nanostructured CuSCN with various morphologies (e.g., NRs and NWs) as HTLs for carbon-based PSCs by tailoring the applied potential and deposition time as depicted in Figure 8a–c [130]. It was found that the applied potential strongly affected the morphologies of CuSCN while nanoplates, NWs and NRs, were formed at an applied potential of −350, −400 and −450 mV. The cell with CuSCN NRs (−450 mV) as HTL exhibited a superior PCE of 12.42% with negligible hysteresis as depicted in Figure 8d, which was much higher than those of CuSCN NWs (10.99%) and CuSCN nanoplates (8.40%) due to the unique morphology, enhanced hole mobility and better energy level alignment. Furthermore, the cell with CuSCN NRs as HTL exhibited much superior stability to those of CuSCN NWs and CuSCN nanoplates under ambient conditions with an RH of >60% at room temperature, as depicted in Figure 8e.

**Figure 9:**
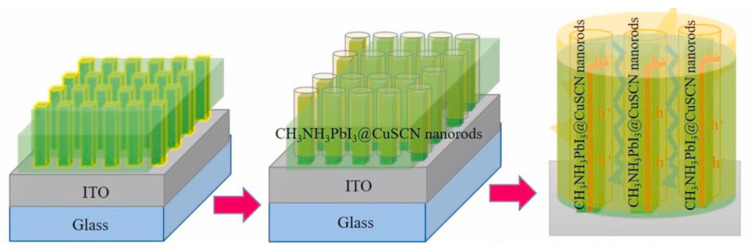
Schematic diagram of the formation of CuSCN@MAPbI_3_ core-shell NRs. Reproduced from [131], with permission from the Organic Electronics, 2021.

Besides the precisely control of applied potential, Ramachandran et al. also optimized the electrochemical deposition method to further boost the PCEs of CuSCN HTL-based PSCs by adding tritionx-100 as surfactants to prepare CuSCN NWs with different morphologies, including void-free NWs and core-shell NWs, as depicted in Figure 9 [131]. It was found that core-shell CuSCN NWs-based PSCs fabricated at an applied potential of −400 mV and a deposition time of 3 min generated a superior PCE of 16.99% with negligible hysteresis, which was much higher than the cell with void-free CuSCN NWs as HTL (10.57%) due to the fact that core-shell CuSCN NRs provided fast channels for charge transfer and suppressed the charge recombination rate. Furthermore, the cell with core-shell CuSCN NRs as HTL maintained 98% of the primary PCE under ambient conditions with an RH of >60%, while CuSCN only retained 87% of the initial PCE under the same conditions.

**Figure 10:**
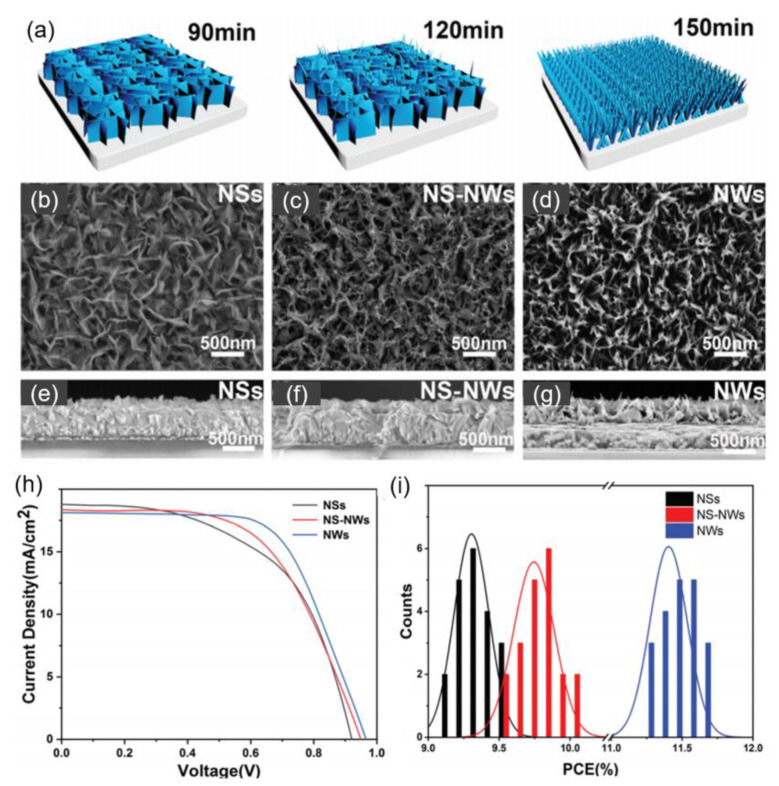
(**a**) Schematic diagrams of the fabrication of NiCo_2_O_4_ HTL with various morphologies at different reaction time; top-view and cross-sectional SEM images of (**b**,**e**) NiCo_2_O_4_ NSs, (**c**,**f**) NiCo_2_O_4_ NSs-NWs, (**d**,**g**) NiCo_2_O_4_ NWs; (**h**) J–V curves and (**i**) PCE histograms of PSCs with NiCo_2_O_4_ NSs, NiCo_2_O_4_ NSs-NWs and NiCo_2_O_4_ NWs as HTLs. Reproduced from [132], with permission from the Dalton Transactions, 2021.

In addition to the morphology control of simple metal oxides (e.g., NiO, WO_3_) and CuSCN as inorganic HTLs for PSCs, Li et al. also synthesized mesoporous complex metal oxides such as NiCo_2_O_4_ nanostructures with different morphologies as HTLs for PSCs by the simple hydrothermal method through tailoring the reaction time [132]. As depicted in Figure 10a–g, NiCo_2_O_4_ NSs, NiCo_2_O_4_ NS-NWs and NiCo_2_O_4_ NWs were obtained by hydrothermal method at reaction time 90, 120 and 150 min, respectively. It was found that NiCo_2_O_4_ NWs HTL-based cell displayed the highest PCE of 11.58% among the three kinds of NiCo_2_O_4_ with different morphologies (Figure 10h,i), which was assigned to the higher conductivity and hole mobility, promoted charge extraction and hole transport capability to reduce the interfacial carrier recombination. In addition, the one-dimensional (1D) structure of NiCo_2_O_4_ NWs provided additional channels for charge transport and suppressed the charge recombination at perovskite/HTL interface. However, it should be noted that the short-circuit current density (*J_sc_*) value of NiCo_2_O_4_ NWs-based cell was lower than the cells based on NiCo_2_O_4_ NSs, NiCo_2_O_4_ NS-NWs HTLs, which was attributed to the larger thickness of NiCo_2_O_4_ NWs film due to the increased hydrothermal reaction time, leading to a reduced light transmittance. Thus, the trade-off between light transmittance and high-performance morphologies should be further investigated to boost the PCEs of NiCo_2_O_4_ HTL-based PSCs.

### 3.4. Nanocomposite Construction

**Figure 11:**
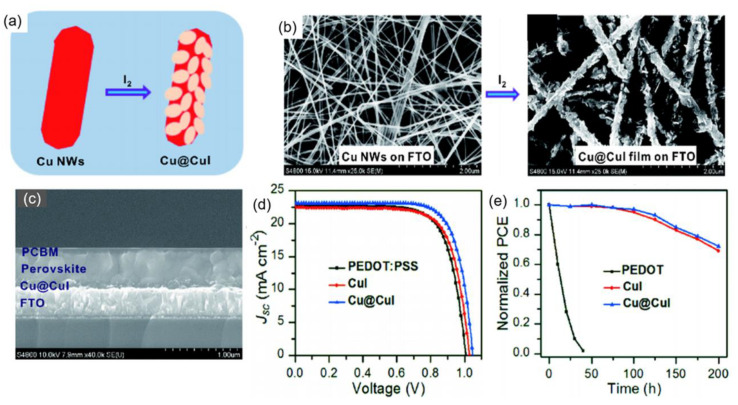
(**a**) Schematic diagram of the transformation of Cu NWs to Cu@CuI nanocomposite in I_2_/ethanol solution; (**b**) top-view SEM image of Cu NWs and Cu@CuI nanocomposite on FTO substrate; (**c**) cross-sectional SEM image of Cu@CuI nanocomposite HTL-based PSC; (**d**) J–V curves and (**e**) ambient stability of PSCs with Cu@CuI, CuI and PEDOT:PSS as HTLs. Reproduced from [133], with permission from the Science China Chemistry, 2019.

As high-performance HTLs for PSCs, single-phase inorganic materials may not fully meet the requirements of HTL, including high conductivity and hole mobility, superior light transmittance and excellent energy level alignment with other layers. [111,115] Thus, nanocomposite construction is regarded as an effective strategy to tackle the above-mentioned problem to achieve high-efficiency and durable inorganic HTL-based PSCs. [133] Up to now, various nanocomposites have been designed and developed as HTLs for PSCs, such as metal/metal iodide, oxide/CuSCN, oxide/metal selenide, metal oxide/oxide and oxide/carbonate, etc. [66,67,133,134,135,136,137]. For instance, Cao et al. synthesized Cu@CuI nanocomposites by an in situ iodination route by using Cu NWs/FTO as the precursors [133]. More specifically, Cu NWs were firstly spin-coated on the FTO substrate, which was further treated in I_2_/ethanol solution for 3 s as depicted in Figure 11a,b. The Cu@CuI nanocomposite HTL-based PSC (Figure 11c) exhibited a superior PCE of 18.8% to those of the cells with CuI (17.5%) and PEDOT:PSS (16.9%) HTLs as depicted in Figure 11d, which was assigned to the boosted extraction and transfer capability of charge carriers to suppress the interfacial recombination. As depicted in Figure 11e, the cell with Cu@CuI nanocomposite HTL maintained ~70% of the original PCE after storing in the air with an RH of 45% for 200 h, while no PCE was retained for the PEDOT:PSS-based cell after a 50 h’s storage under the same conditions. Cao et al.’s study represents a new in situ nanocomposite construction strategy for the fabrication of high-efficiency and durable inorganic HTL-based PSCs.

In addition to metal/metal iodide, oxide/CuSCN and oxide/metal selenide nanocomposites have also been designed and developed as inorganic HTLs for PSCs [134,135,136]. For instance, Lee et al. prepared CuCaO_2_ NPs/CuSCN as the nanocomposite HTL to boost the PCE and stability of PSCs [134]. Firstly, CuCaO_2_ NPs were modified by amino silane to reduce the accumulation to obtain a uniform NPs-based film. After this, CuSCN solution was then coated on CuCaO_2_ NPs-based film while CuSCN was penetrated into CuCaO_2_ NPs-based film, which was beneficial to reducing the roughness of CuCaO_2_ NPs-based film and improving the hole extraction capability of CuCaO_2_ NPs-based HTL. As a result, the cell with CuCaO_2_ NPs/CuSCN nanocomposite HTL generated a superb PCE of 16.7%, which was 8% higher than that of the CuSCN HTL-based cell due to the remarkably reduced trap density (40%). Furthermore, the thermal and moisture stability of CuCaO_2_/CuSCN nanocomposite HTL-based device has also significantly improved. The PCE of the CuCaO_2_/CuSCN-based cell still maintained 85% of its original PCE after 400 h’s storage at 85 °C and 85% RH. More recently, Kim et al. successfully synthesized Cu_2_O-CuSCN nanocomposite by using a facile one-step deposition route to boost the PCE and stability of CuSCN-based PSCs [135]. The Cu_2_O–CuSCN nanocomposite-based cell generated a higher PCE of 19.2% than that of the CuSCN-based device (17.7%) due to the improved hole mobility and significantly reduced trapping sites. The Cu_2_O–CuSCN nanocomposite-based PSC still retained 90% of the initial PCE after storing in harsh conditions (85 °C and 85% RH) for 720 h due to the much-reduced interface degradation between perovskite layer and CuSCN HTL. Ramachandran et al. fabricated various CuSCN/CuI nanocomposite-based HTLs by electrodeposition method through tailoring the electrochemical potential and deposition time for carbon-based PSCs [136]. The cell based on the optimized CuSCN/CuI nanocomposite HTL with a thickness of 142 nm (electrochemical potential of −400 mV and deposition time of 2 min) presented a superb PCE of 18.82%, which was much higher than the CuSCN HTL-based device (16.66%) due to the enhanced light transmittance, improved surface morphology and enlarged crystal sizes of PSCs.

**Figure 12:**
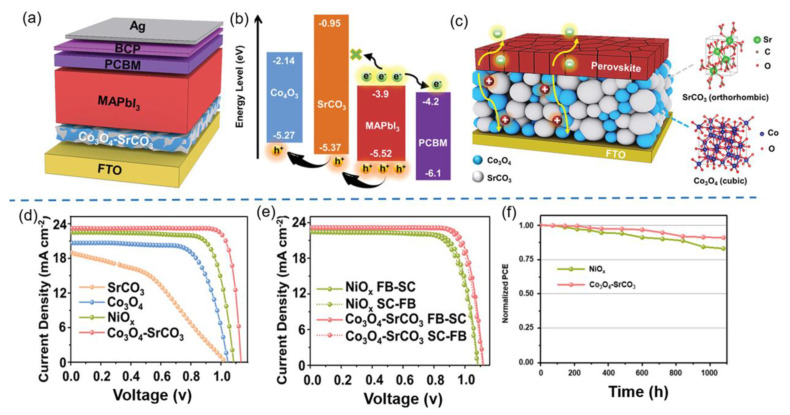
(**a**) Structural and (**b**) energy level diagrams of Co_3_O_4_-SrCO_3_ HTL-based PSC; (**c**) schematic diagram of charge transfer pathways in Co_3_O_4_-SrCO_3_ HTL-based PSC; (**d**) J–V curves of PSCs with SrCO_3_, Co_3_O_4_, Co_3_O_4_-SrCO_3_ and NiO_x_ as HTLs; (**e**) J–V curves of PSCs with Co_3_O_4_-SrCO_3_ and NiO_x_ as HTLs under forward and reverse scan directions; (**f**) ambient stability of PSCs with Co_3_O_4_-SrCO_3_ and NiO_x_ as HTLs. Reproduced from [67], with permission from the Advanced Functional Materials, 2019.

The rational design of nanocomposite HTL may also extend the light absorption range of PSCs from 300–800 nm to 800–1000 nm to remarkably boost the PCEs of PSCs [66]. In 2020, Davood et al. designed core-shell structured NiO@GeSe (25 nm for core and 50 nm for shell) nanocomposite as HTLs for PSCs [66]. As the shell, GeSe displayed an absorption spectrum of 800–1000 nm, which was beneficial to extending the light absorption range of pristine PSCs. As the core, NiO provided fast channels for hole transport to improve the hole mobility and reduce the carrier recombination. Consequently, the NiO@GeSe HTL-based cell with optimized film thickness of 120 nm produced an excellent PCE of 20.29%, which was 40% higher than the cell with GeSe NRs HTL (14.53%). In addition to NiO@GeSe core-shell HTL, oxide/oxide core-shell HTLs have also been constructed to boost the PCEs of NiO-based PSCs. Farzaneh et al. synthesized core-shell CuO@NiO nanospheres by a solvothermal route for the application as HTLs for PSCs [137]. The core-shell CuO@NiO-based cell generated a higher PCE of 10.11% than that of the pristine NiO-based device (8.58%), which was mainly due to the better coverage of CuO NPs over NiO nanospheres to reduce the cracks of NiO nanospheres, improved conductivity and the reduced non-radiative recombination at perovskite film/HTL interface. Very recently, Ge et al. synthesized Co_3_O_4_-SrCO_3_ nanocomposite by heat treatment as HTLs for PSCs, as depicted in Figure 12a [67]. As depicted in Figure 12b,c, in such nanocomposite, Co_3_O_4_ with a narrow band gap was used to improve the hole migration rate while SrCO_3_ with a wide band gap was employed to selectively block the electrons and reduce carrier recombination. Furthermore, a cross-gap type I heterojunction was formed in Co_3_O_4_-SrCO_3_ nanocomposite with uniform and compact morphology, which effectively enhanced the conductivity and hole mobility of HTL as well as reduced the charge recombination between the perovskite film and HTL. Consequently, MAPbI_3_-based PSC with Co_3_O_4_-SrCO_3_ nanocomposite HTL produced a superb PCE of 21.84%, which was much higher than the cells with Co_3_O_4_ (15.47%), SrCO_3_ (8.08%) and NiO_x_ (18.41%) as HTLs (Figure 12d,e). As depicted in Figure 12f, the optimized Co_3_O_4_-SrCO_3_ nanocomposite-based unencapsulated cell maintained 91% of the original PCE after 1000 h’s storage under ambient conditions with an RH of 15%, much superior to the NiO-based cell (a PCE retention ratio of ~80% under the same conditions).

### 3.5. Crystal Structure Regulation and Bilayer HTL Design

**Figure 13:**
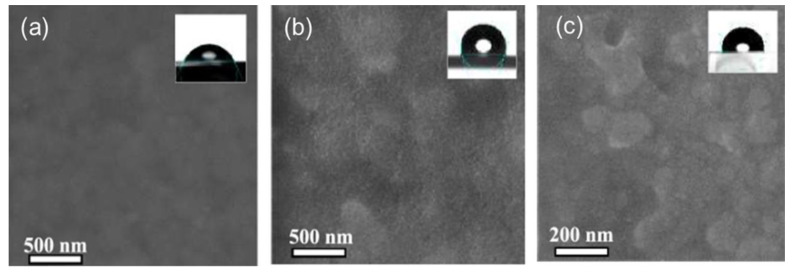
Top-view SEM image of various HTLs: (**a**) Spiro-OMeTAD, (**b**) wurtzite-CTS and (**c**) zincblende-CTS with water contact angles on the HTL surface shown inset. Reproduced from [138], with permission from the ACS Applied Energy Materials, 2021.

In addition to the above-mentioned strategies for the design and fabrication of inorganic HTLs, crystal structure regulation and bilayer HTL design have also been reported to boost the PCE and stability of inorganic HTL-based PSCs, although the relevant literature is limited until now. For instance, Heidariramsheh et al. successfully synthesized Cu_2_SnS_3_ (CTS) NPs with different crystal structures (zincblende and wurtzite) as HTLs for PSCs [138]. More specifically, zincblende CTS and wurtzite CTS were synthesized by the oil amine solvent heating method using sulfur and thiourea as sulfur sources, respectively. The CTS NPs were then dispersed in non-polar chloroform solution to obtain CTS inks, which were spin-coated on perovskite layer as HTL. As depicted in Figure 13, Spiro-OMeTAD HTL displayed has the smoothest surface while zincblende-CTS exhibited the roughest surface, which was due to the NPs agglomeration in the HTL inks. In addition, the wurtzite-CTS and zincblende-CTS displayed much better hydrophobic capability than that of Spiro-OMeTAD due to the addition of lithium salts in Spiro-OMeTAD, which may be beneficial for the achievement of high moisture tolerance. The cell with wurtzite-CTS HTL exhibited a higher PCE of 13.1% than that of zincblende-CTS-based cell (7.87%), which was assigned to the more uniform and flattened wurtzite-CTS film, monodispersed wurtzite-CTS NPs and better energy level alignment. As for the moisture stability, the cell with wurtzite-CTS HTL retained ~90% of the primary PCE after 1200 h’s storage under ambient conditions with an RH of >45%, which was much better than that of Spiro-OMeTAD-based cell with a PCE retention ratio of ~60% under the same conditions due to the remarkably enhanced hydrophobic capability of wurtzite-CTS HTL.

**Figure 14:**
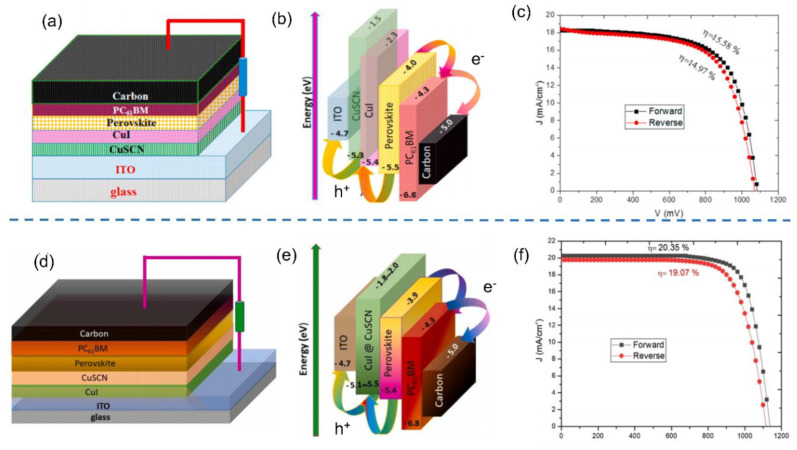
(**a**) Structural diagram and (**b**) energy levels and (**c**) J–V curves under reverse and forward scan directions of carbon-based inverted PSCs with bilayer CuSCN/CuI HTL. Reproduced from [139], with permission from the Journal of Alloys and Compounds, 2021. (**d**) Structural diagram and (**e**) energy levels and (**f**) J–V curves under reverse and forward scan directions of carbon-based inverted PSCs with bilayer CuI/CuSCN HTL. Reproduced from [140], with permission from the Ceramics International, 2021.

As mentioned above, CuSCN and CuI have been extensive as inorganic HTLs for PSCs [133,134,135]. CuSCN displayed a wide band gap of 3.4–3.9 eV and low hole mobility of 0.1 cm^2^ V^−1^ s^−1^, while CuI exhibited a narrow band gap of 3.1 eV and superior hole mobility of 40 cm^2^ V^−1^ s^−1^. [133,135] Thus, it is crucial to fully utilize the merits of CuSCN and CuI to further boost the PCEs of inorganic HTL-based PSCs. In 2021, Ramachandran et al. fabricated nanostructured CuSCN/CuI bilayer HTL by electrodeposition method for carbon-based inverted PSCs [139]. The PSC with CuSCN/CuI bilayer HTL (Figure 14a,b) generated a high PCE of 15.58% (Figure 14c) due to the improved energy level alignment, increased hole mobility and reduced sheet resistance. Furthermore, the cell with CuSCN/CuI bilayer HTL maintained 85% of the original PCE after aging under ambient conditions with RH of >60% for 1400 h. In addition, Ramachandran et al. further optimized the performance of bilayer HTL-based carbon-based inverted PSCs by tailoring the positions of CuSCN and CuI layers as well as modifying the electrodeposition method through controlling the electrochemical potential and deposition time to achieve the optimal thickness and transparency [140]. After optimizing electrochemical potential (−400 mV) and deposition time (2 min), the carbon-based PSC with CuI/CuSCN bilayer HTL (Figure 14d,e) produced a superior PCE of 20.35% (Figure 14f) to that of PSC with CuI-CuSCN composite HTL (18.76%). Furthermore, the cell with CuI/CuSCN bilayer HTL retained 90% of the original PCE after a storage under ambient conditions with RH of >60% for 1400 h.

## 4. Conclusions and Perspectives

As a key component in PSCs, high-performance HTLs should meet several requirements, including high light transmittance, hole extraction capability and hole mobility, superior moisture and thermal stability, proper energy level alignment with the perovskite layer and low cost. At present, organic HTLs have been widely used in PSCs, showing superior PCEs. Nevertheless, organic HTLs suffered from poor stability and high cost, which have become one of the largest obstacles to the large-scale applications of PSCs. As an important alternative to organic HTLs, nanostructured inorganic materials have also received increasing attention as HTLs for PSCs, which not only significantly improved the moisture/thermal stability but also reduced the raw material costs of PSCs. In this review, the recent advances in the development and fabrication of nanostructured inorganic HTLs for PSCs are reviewed by emphasizing the superiority of nanostructured inorganic HTLs over organic counterparts. In addition, several strategies to boost the PCE and stability of inorganic HTLs are also proposed and discussed.

Table 1 lists the performance comparison of PSCs with nanostructured inorganic HTLs designed by functional/selectively doping. As can be observed, functional/selectively doping is widely employed to boost the hole mobility of NiO_x_ and the PCEs of corresponding PSCs. Among various cations, Sm^3+^ doping is reported to be highly effective in boosting the PCEs of NiO_x_-based PSCs. A highly attractive PCE of 20.71% was achieved by Sm:NiO_x_-based PSC, which was assigned to the unique electronic properties of trivalent Sm^3+^ cations with partially filled 4f orbits and empty 5d orbits, significantly reducing the formation energy of Ni vacancies and improving the conductivity. Table 2 shows the performance comparison of PSCs with nanostructured inorganic HTLs with different morphologies. For example, nanostructured CuSCN HTLs with different morphologies (e.g., NWs, NRs) are obtained by adjusting the parameters of electro-deposition method, such as the deposition time and applied potential. Among various CuSCN HTLs with different morphologies, CuSCN NWs-based PSCs exhibited the highest PCE due to the formation of core-shell CuSCN/MAPbI_3_ NRs during the perovskite film deposition process, which effectively enhanced the hole extraction/transport capability and increased the interaction between perovskite film and HTL. Table 3 depicts the performance comparison of PSCs with inorganic nanocomposite HTLs. As can be seen, the PSC with Co_3_O_4_-SrCO_3_ nanocomposite HTL produced the highest PCE of 21.84%, which was mainly attributed to the improved hole migration rate by Co_3_O_4_ and selectively electron-blocking capability by SrCO_3_.

Although numerous efforts have been devoted to the development and fabrication of nanostructured inorganic HTL-based PSCs during the past 5 years, some challenges and problems still remain, such as low conductivity, NPs aggregation in the HTL ink and thick nanostructured inorganic HTL. Thus, the design of new materials and fabrication routes are crucial for the further performance enhancement of nanostructured inorganic HTL-based PSCs in the future. As the low conductivity, trivalent lanthanide metal ions have been proved to be effective in enhancing the conductivity of NiO_x_. However, such enhancement is limited due to the low doping amount of lanthanide cations and the fixed atomic environment of simple oxides. Perovskite oxides (ABO_3_) with fully/mostly occupied lanthanide cations at A-site, such as nanostructured SmCoO_3_, should be highly attractive as inorganic HTLs for PSCs. In addition, various perovskite oxides can be designed to achieve desired performance as HTLs for PSCs due to their excellent compositional and structural flexibility. As for the challenges of NPs aggregation and thick HTL film, some advanced deposition routes, such as atomic layer deposition (ALD), can be utilized to fabricate nanofilm-based inorganic HTLs for PSCs [141]. More specifically, ALD is conducive to the formation of nanofilms with uniform morphology and controllable thickness due to the self-limiting growth mechanism of ALD. Besides the above existing problems, the hole extraction/transport mechanism of nanostructured inorganic HTLs still remains unclear, which requires further investigations in the future (e.g., advanced characterization techniques and theoretical calculations).

## Figures and Tables

**Table 1:** Performance comparison of PSCs with doped NPs-based inorganic HTLs.

HTL	Cell Configuration	Hole Mobility (cm^2^ V^−1^ s^−1^)	PCE (%)	Ref
Co-NiO NPs	ITO/Co-NiO NPs/MAPbI_3_/PCBM/Ag	/	14.50	[121]
Fe-NiO_x_ NPs	ITO/Fe-NiO_x_ NPs/MAPbI_3_/PCBM/BCP/Ag	6.22	17.57	[122]
Ni^3+^-NiO NSs	FTO/Ni^3+^-NiO NSs/FA_0.83_MA_0.17_PbI_2.49_Br_0.51_/PCBM/ZnO/Al	/	17.75	[117]
Cu-NiO_x_ NPs	ITO/Cu-NiO_x_ NPs/MAPbI_3_/PCBM/BCP/Ag	1.05 × 10^−2^	18.58	[63]
Cu:NiO NPs	ITO/Cu:NiO NPs/MAPbI_3_/C_60_/BCP/Ag	2.53	20.15	[123]
Sm:NiO_x_	ITO/Sm:NiO_x_/FA_0.92_Cs_0.08_PbI_3_	/	20.71	[124]
Cu_3_SbS_4_ NCs	FTO/c-TiO_2_/m-TiO_2_/Cs_0.0475_FA_0.7885_MA_0.1615_PbI_2.49_Br_0.51_/Cu_3_SbS_4_ NCs/Au	/	13.00	[126]
CuIn_0.5_Ga_0.5_S_2_ NPs	FTO/TiO_2_/Cs_0.0475_FA_0.7885_MA_0.1615_ PbI_2.49_ Br_0.51_/CuIn_0.5_Ga_0.5_S_2_ NPs/Au	0.4 × 10^−3^	15.58	[127]

**Table 2:** Performance comparison of PSCs based on nanostructured inorganic HTLs with different morphologies.

HTL	Cell Configuration	Ambient Stability (PCE Retention Ratio)	PCE (%)	Ref
CuSCN NRs	ITO/CuSCN NRs/MAPbI_3_/PCBM/Carbon	80% after 1200 h	12.42	[130]
CuSCN hexagonal prisms	ITO/CuSCN hexagonal prisms/MAPbI_3_/C_60_/Bphen/Ag	/	11.40	[69]
CuSCN NWs	ITO/CuSCN NWs/MAPbI_3_/PC_61_BM/Carbon	~100% after 1272 h	16.99	[131]
NiCo_2_O_4_ NWs	ITO/NiCo_2_O_4_ NWs/MAPbI_3_/PC_61_BM/Ag	80% after 240 h	11.58	[132]
WO_3_ NSs	ITO/Nafion modified-ZnO/MAPbI_3_/P3HT:PCBM/WO_3_ NSs/Ag	~100% after 240 h	7.76	[129]

**Table 3:** Performance comparison of PSCs with typical nanocomposite inorganic HTLs.

HTL	Cell Configuration	Ambient Stability (PCE Retention Ratio)	PCE (%)	Ref
Cu@CuI	FTO/Cu@CuI/CsFAMAPb(BrI)_3_	70% after 200 h	18.8	[133]
CuCaO_2_–CuSCN	ITO/SnO_2_/Cs_0.0475_FA_0.7885_MA_0.1615_PbI_2.49_ Br_0.51_/CuCaO_2_-CuSCN/Au	80% after 400 h	16.70	[134]
Cu_2_O–CuSCN	ITO/SnO_2_/Cs_0.0475_FA_0.7885_MA_0.1615_PbI_2.49_ Br_0.51_/Cu_2_O-CuSCN/Au	>90% after 720 h	19.20	[135]
CuSCN/CuI	ITO/CuSCN/CuI/MAPbI_3_/PCBM/Carbon	95% after 1400 h	18.82	[136]
NiO@GeSe	ITO/TiO_2_/NiO@GeSe/MAPbI_3_/CuSCN/Au	/	20.29	[66]
Co_3_O_4_–SrCO_3_	FTO/Co_3_O_4_-SrCO_3_/MAPbI_3_/PCBM/BCP/Ag	~100% after 300 h	21.84	[67]

## Data Availability

The systematic review data used to support the findings of this study are included with the article.
